# Regulation of anoikis by extrinsic death receptor pathways

**DOI:** 10.1186/s12964-023-01247-5

**Published:** 2023-09-04

**Authors:** Ying-Hao Han, Yuan Wang, Seung-Jae Lee, Mei-Hua Jin, Hu-Nan Sun, Taeho Kwon

**Affiliations:** 1 College of Life Science & Biotechnology, Heilongjiang Bayi Agricultural University, Daqing, 163319 China; 2 Functional Biomaterial Research Center, Korea Research Institute of Bioscience and Biotechnology (KRIBB), Jeonbuk, 56212 Republic of Korea; 3 Department of Applied Biological Engineering, KRIBB School of Biotechnology, University of Science and Technology, Daejeon, 34113 Republic of Korea; 4 Primate Resources Center, Korea Research Institute of Bioscience and Biotechnology (KRIBB), Jeonbuk, 56216 Republic of Korea; 5 Department of Functional Genomics, KRIBB School of Bioscience, University of Science and Technology, Daejeon, 34113 Republic of Korea

**Keywords:** Aanoikis, Death receptor pathway, Fas, TNFR1/TNFR2, DR4/DR5, Cancer metastasis

## Abstract

Metastatic cancer cells can develop anoikis resistance in the absence of substrate attachment and survive to fight tumors. Anoikis is mediated by endogenous mitochondria-dependent and exogenous death receptor pathways, and studies have shown that caspase-8-dependent external pathways appear to be more important than the activity of the intrinsic pathways. This paper reviews the regulation of anoikis by external pathways mediated by death receptors. Different death receptors bind to different ligands to activate downstream caspases. The possible mechanisms of Fas-associated death domain (FADD) recruitment by Fas and TNF receptor 1 associated-death domain (TRADD) recruitment by tumor necrosis factor receptor 1 (TNFR1), and DR4- and DR5-associated FADD to induce downstream caspase activation and regulate anoikis were reviewed. This review highlights the possible mechanism of the death receptor pathway mediation of anoikis and provides new insights and research directions for studying tumor metastasis mechanisms.

Video Abstract

**Supplementary Information:**

The online version contains supplementary material available at 10.1186/s12964-023-01247-5.

## Background

**Fig. 1:**
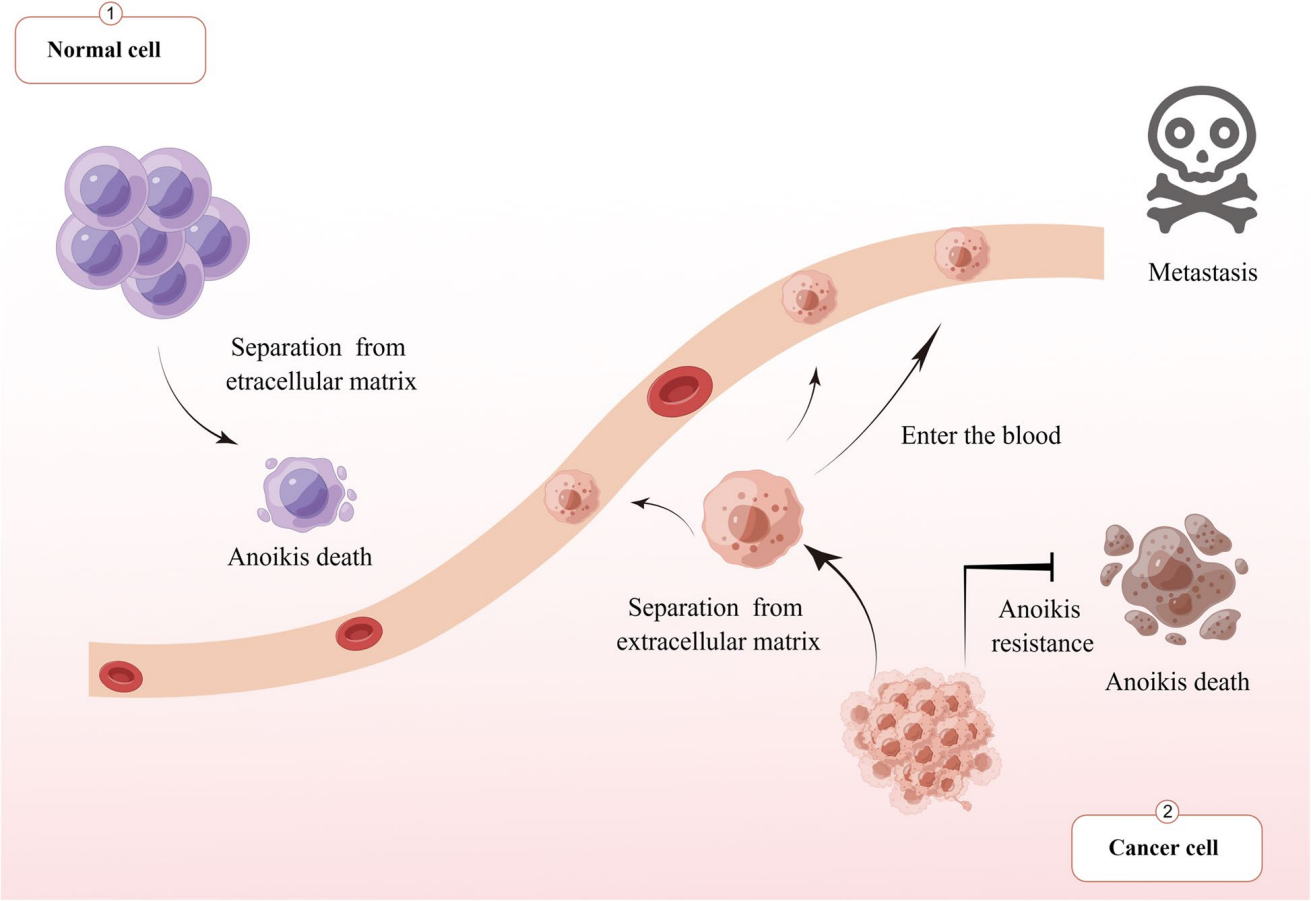
Cell fate of normal cells and metastatic tumor cells after losing adhesion to the extracellular matrix. When normal cells lose their adhesion to the extracellular matrix, apoptosis occurs because the body prevents the wrong connection of cells. However, after losing adhesion to the extracellular matrix due to its metastasis characteristics, tumor cells develop resistance, survive without adhesion to the extracellular matrix, and can flow to other tissues and organs through blood and lymph for adhesion and metastasis

Cancer cells spread from the primary to distant sites through the blood and lymph in a multi-step process that includes separation from the primary site, entry into the blood and lymph, passage through circulation, attachment to distant target organs by extravasation, and survival [1]. Once cells enter the blood and lymph and start the cycle, they lack the appropriate extracellular matrix (ECM) attachment that acts as a barrier to metastasis [2]. Cell death usually occurs after cells lose contact with neighboring cells or the ECM. This type of cell death is called anoikis [3], a special form of cell escape death that differs from other forms of death [4]. To acquire the ability to transfer, tumor cells must establish anoikis resistance and survive without adhering to the ECM [5]. Anoikis plays an important physiological role by regulating cell homeostasis in tissues [6] and is critical in tumor metastasis, development, tissue remodeling, and wound-healing responses [7–9] (Fig. 1).

Anoikis is affected by various pathways [10] and reportedly causes cell death due to loss of cell adhesion and ECM separation [11]. Anoikis, necrosis, ferroptosis, pyroptosis, and apoptosis are different forms of cell death, involving distinct signaling pathways and molecular mechanisms. Necrosis is an irregular and passive form of cell death, typically caused by external factors such as trauma, infection, or ischemia [12]. Necrosis is characterized by cell membrane rupture and leakage of cellular contents, without the requirement of caspase proteinase activation. During necrosis, cellular metabolism and energy production cease, resulting in cellular structural damage and death [13]. Ferroptosis typically occurs due to the excessive accumulation of intracellular iron ions, leading to increased oxidative stress within the cell. This form of cell death involves aberrant regulation of iron ion transport, storage, and metabolism, resulting in oxidative damage and cell death. The signaling pathways of ferroptosis have been found to involve proteins such as iron ion regulators, the production of reactive oxygen species (ROS), and protein kinases associated with cell death [14, 15]. Pyroptosis is an inflammatory form of cell death, usually occurring in response to infection or abnormal intracellular signaling. It is characterized by cell membrane rupture and release of cellular contents, accompanied by the activation of intracellular inflammatory factors and the occurrence of inflammatory reactions. The signaling pathway of pyroptosis involves the formation of inflammasomes and the activation of inflammatory-related proteinase caspase-1. The activation of these proteinases leads to the release of inflammatory factors and the occurrence of inflammatory reactions, ultimately resulting in cell death [16]. Apoptosis is a highly regulated and active form of cell death, involving both intrinsic and extrinsic pathways. The intrinsic pathway primarily involves the release of mitochondria and the activation of caspases, while the extrinsic pathway mainly involves the activation of cell membrane receptors and caspases. These signaling pathways ultimately lead to the activation of caspase proteinases within the cell, triggering a cascade of events associated with apoptosis, including DNA fragmentation, nuclear fragmentation, and cell membrane rupture, ultimately leading to cell death [17]. Anoikis is a caspase-dependent form of cell death, indicating that cells undergoing anoikis activate caspase family proteinases, triggering a cascade of events leading to cell death, similar to apoptosis. However, anoikis, which is caused by the loss of contact with the extracellular matrix (ECM), exhibits some unique features in terms of cell signaling. It involves several major signaling pathways, including integrin signaling, PI3K-AkT signaling, and Fas signaling [3]. The activation of these pathways leads to the activation of caspases associated with apoptosis, ultimately resulting in cell death. Anoikis primarily occurs in non-tumor cells that lose contact with the ECM, while apoptosis is also common in tumor cells. Integrins are highly abundant and important cell surface adhesion receptors expressed in all cell types except red blood cells in mammals [18]. These receptors are composed of alpha- and beta-subunits, which associate to form heterodimer molecules that span the cell membrane. They play a crucial role in facilitating cell interactions with molecules present in the ECM [19]. Integrins are key regulators of numerous physiological processes, encompassing cell migration, survival, proliferation, and gene expression. These multifunctional receptors play a pivotal role in orchestrating various cellular events, ensuring cellular homeostasis and tissue integrity [20–22], and differ from other receptors in their ability to transmit signals in both directions [23]. Signals within cells can originate from priming receptors that activate this phenomenon [24]. The first downstream component activated by integrin is focal adhesion kinase (FAK) [19]. Autophosphorylation of FAK leads to the activation of Src family kinases and activation of the PI3K-AkT and Raf-MEK-ERK signaling pathways [25, 26]. Integrins thus regulate almost every aspect of the behavior of adherent cell types, from cell proliferation and survival to migration and invasion [27]. Studies have shown that inhibition of caspase-8 can increase anoikis resistance in colon cancer cells; however, inhibition of caspase-9 activity does not achieve the same results. Thus, the caspase-8-mediated extrinsic pathway is more sensitive to anoikis than the intrinsic pathway [28]. In other words, death receptor-mediated apoptosis signals are more sensitive to the control of anoikis than Fak-mediated survival signals, which can explain why the loss of anchoring leads to an increase in Fas expression and Fas-ligand (Fas-L) expression during the anoikis of tumor cells [29]. Recent studies have also indicated the involvement of FAS/CD95 expression in the control of inflammatory signaling pathways in triple-negative breast cancer (TNBC) [30, 31]. This suggests that besides its established function as an apoptosis-inducing receptor, CD95/Fas also possesses various non-apoptotic activities. In the context of TNBC inflammation mediated by Fas/CD95, it has been reported that CD95 can interact with the Kip1 ubiquitination-promoting complex protein 2 (KPC2) independently of CD95L. This interaction has been associated with NF-κB suppression. Similarly, in Fas/CD95-mediated glioblastoma cells, a CD95L-independent CD95 signaling pathway has been identified, which contributes to the maintenance of malignant characteristics in glioma-initiating cells [32]. Our manuscript also describes the findings from other studies that show an increase in apoptosis caused by CD95/Fas independent of its ligand. These phenomena are thought to be explained by the involvement of endocytosis. Membrane trafficking of death receptors plays a crucial role in the transmission of Fas-mediated apoptotic signals, and the effective formation of the death-inducing signaling complex (DISC) occurs at the nuclear/cytoplasmic level, requiring internalization of Fas receptors [33]. Moreover, depending on the cell type and environment, non-apoptotic signaling pathways such as the activation of the transcription factor NF-κB can also be activated upon ligand binding to Fas. It has been observed that CD95 interacts with the KPC2 protein, promoting partial degradation of p105 (NF-kB1), leading to the generation of p50 homodimers, thereby inhibiting NF-κB-driven gene expression. The specific mechanism involves the interaction of KPC2 with the C-terminal region of CD95 and the recruitment of RelA (p65) and KPC1 as adapters, where KPC1 functions as an E3 ubiquitin-protein ligase promoting the degradation of p105 into p50. Loss of CD95 results in the release of KPC2, limiting the formation of inhibitory NF-κB homodimer complexes (p50/p50), thereby promoting NF-κB activation and the production of pro-inflammatory cytokines [30]. There are also studies show that an endogenous inhibitor of Fas-mediated signaling, FLICE inhibitory protein (Flip), is significantly downregulated, and anoikis may be involved in upregulating the death receptor and its ligand [1, 29, 34]. The external pathway mediated by death receptors may be an inhibitor of anoikis and a promising target for anti-metastasis therapy strategies. Therefore, this review focuses on the possible role of death receptor-mediated external pathways in anoikis to further supplement the death receptor pathway in anoikis.

### Initiation form of anoikis

**Fig. 2:**
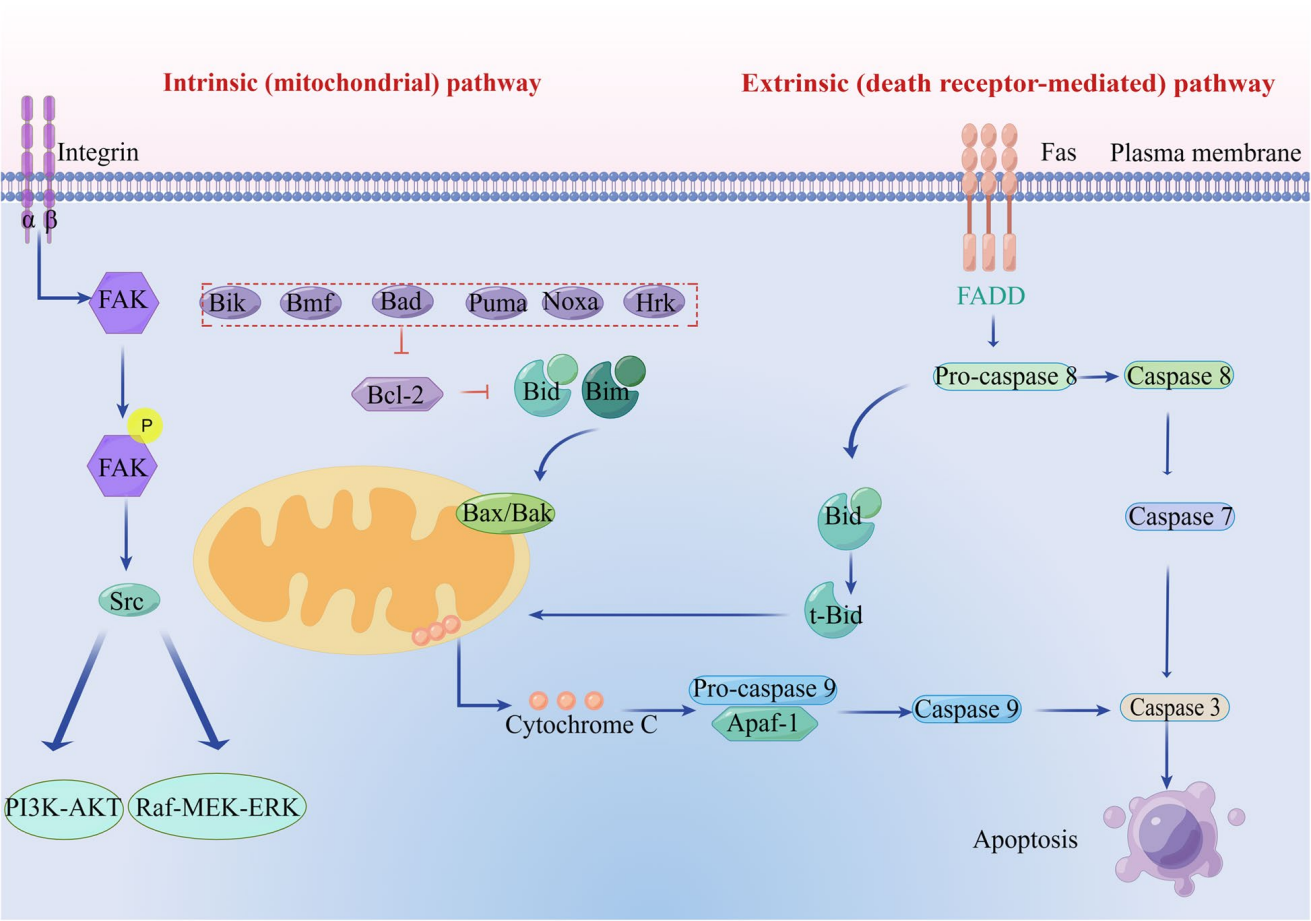
Pathway diagram of internal and external mechanisms of anoikis. Lack of ECM exposure or inappropriate ECM exposure fails to activate pro-survival signaling, leading to a reduction in the anti-apoptotic pathway and, thus, activation of extrinsic death receptors and intrinsic integrin-mediated mitochondria-dependent anoikis. The death receptor pathway triggers two different extrinsic pathways in different cell types: type 1 leads to direct proteolysis of the target dependent on caspase-7 activation; type 2, which aggregates in the intrinsic pathway through a truncated form of Bid (tBid), promotes the release of mitochondrial cytochrome c and assembly of apoptotic corpuscles. These events induce proteolysis of caspase-specific targets, thereby promoting anoikis

Initiation and execution of anoikis are mediated by caspase-dependent apoptosis [35] and may prevent ectopic cell growth in inappropriate body parts [36]. When ECM exposure is lacking or inappropriate, it fails to activate pro-survival signaling [37], resulting in reduced anti-apoptotic pathways that activate death receptors and mitochondria-dependent anoikis [38]. In general, anoikis is regulated by both endogenous and exogenous cell death pathways [39]. The activation of endogenous pathways involves the upregulation of pro-apoptotic molecules, including Bad, Bik, Puma, Hrk, Bmf, and Noxa. These molecules play a crucial role in inducing apoptosis by counteracting the anti-apoptotic proteins within the Bcl-2 family. Additionally, intracellular signals triggered by DNA damage or cytokine deprivation contribute to the modulation of these pathways, further enhancing the apoptotic response, Bcl-2 inhibits pore formation in mitochondria of Bad and Bax, also inhibits apoptosis by inhibiting Bim, the activator of apoptosis. These events induce proteolysis of caspase-specific targets, thereby promoting anoikis [36, 40]. Cell surface death receptors such as Fas/CD95, tumor necrosis factor receptor 1 (TNFR1), DR4, and DR5 are involved in exogenous pathways [41]. External pathways are also activated by the increased expression of Fas receptors [42]. Fas receptor activation triggers two distinct external pathways [43]: type I, leading to direct proteolysis of the target dependent on caspase 7 activation. This type I cell line is directly induced by caspase-8 activation and thus induces apoptosis of the caspase cascade reaction. In type II cells, death-inducing signal complex (DISC) formation differs from that in type I cells because it is strongly inhibited, and caspase-8 is activated only in a small amount [44, 45]. A small amount of active caspase-8 can shear the pro-apoptotic protein molecule Bid and transpose it to the mitochondria, and activated caspase-8 is added to split the cytoplasmic Bid into two fragments [46]. Namely, the 15 kDa large fragment truncated Bid (tBid) and 13 kDa small fragment (tBid) are translocated into the mitochondria to induce apoptosis, while the small fragment remains in the cytoplasm [47], tBid, a truncated form of Bid protein, undergoes translocation from the cytoplasm to the mitochondria. Once localized in the mitochondria, tBid interacts with other Bcl-2 family proteins, particularly through its BH3 domain. This interaction induces the release of chromosomal turbulence, leading to perturbations in chromosomal stability and function [47]. When tBid is transferred into the mitochondria, the Bcl-2 family apoptosis-inducing proteins Bax and Bak undergo conformational changes and form oligomers [48]. Oligomeric Bax and Bak form mitochondrial membrane channels and promote the release of the apoptotic protein cytochrome c. Furthermore, tBid exhibits the ability to bind to anti-apoptotic proteins such as Bcl-2 and Bcl-xl. By doing so, tBid impedes their inhibitory effect on the oligomerization of Bax and Bak, thereby promoting apoptosis. This interaction also leads to alterations in mitochondrial membrane potential and facilitates the release of cytochrome c into the cytoplasm. In the cytoplasm, cytochrome c forms a complex with Apaf1 (a factor associated with apoptosis) and pro-caspase-9, leading to the autocatalytic cleavage of pro-caspase-9 into an active form, and then activated caspase-9 reactivates the downstream effector enzymes caspase-3, casepase-6, and caspase-7 [49] (Fig. 2).

### Construction of anoikis model

#### Ultra-low adhesion plate culture

Anoikis is induced by growing cells on ultra-low adhesion plates (Corning), as described previously [50, 51].

#### Anoikis is induced by the changing fibronectin matrix

Cells are treated with recombinant fibronectin (FN), which has a mutated domain that alters the fibronectin matrix, causing it to malfunction and induce anoikis [52, 53].

#### Anoikis caused by drug treatment

### H_2_O_2_

The cells were cultured in a medium containing H_2_O_2_ for 14 days, during which the medium was replaced with a newly formulated H_2_O_2_ medium every 2 days. After 14 days, adherent cells were inoculated into poly-hema coated plates to simulate anoikis [54].

### Tunicamycin

Tunicamycin is a substance that induces endoplasmic reticulum stress by blocking the protein N-glycosylation process. This can cause an accumulation of misfolded proteins, leading to protein aggregation and ultimately, cell death through a process called anoikis [55, 56].

### Thapsigargin

Thapsigargin is a compound that releases calcium ions from the endoplasmic reticulum into the cytoplasm. This process can contribute to oxidative stress and may also play a role in the regulation of anoikis [57, 58].

### Staurosporine

Staurosporine is an alkaloid that was first isolated from Streptomyces staurosporeus in 1977. It is a non-specific inhibitor of various kinases and has been shown to induce apoptosis in many types of cancer cells, including cervical cancer. In a graduate study exploring the antitumor effects of staurosporine in the cervical cancer microenvironment, it was discovered that this alkaloid also induced significant anoikis in single-cell suspensions [59, 60].

### Tumornecrosisfactor-alpha (TNF-α)

**Fig. 3:**
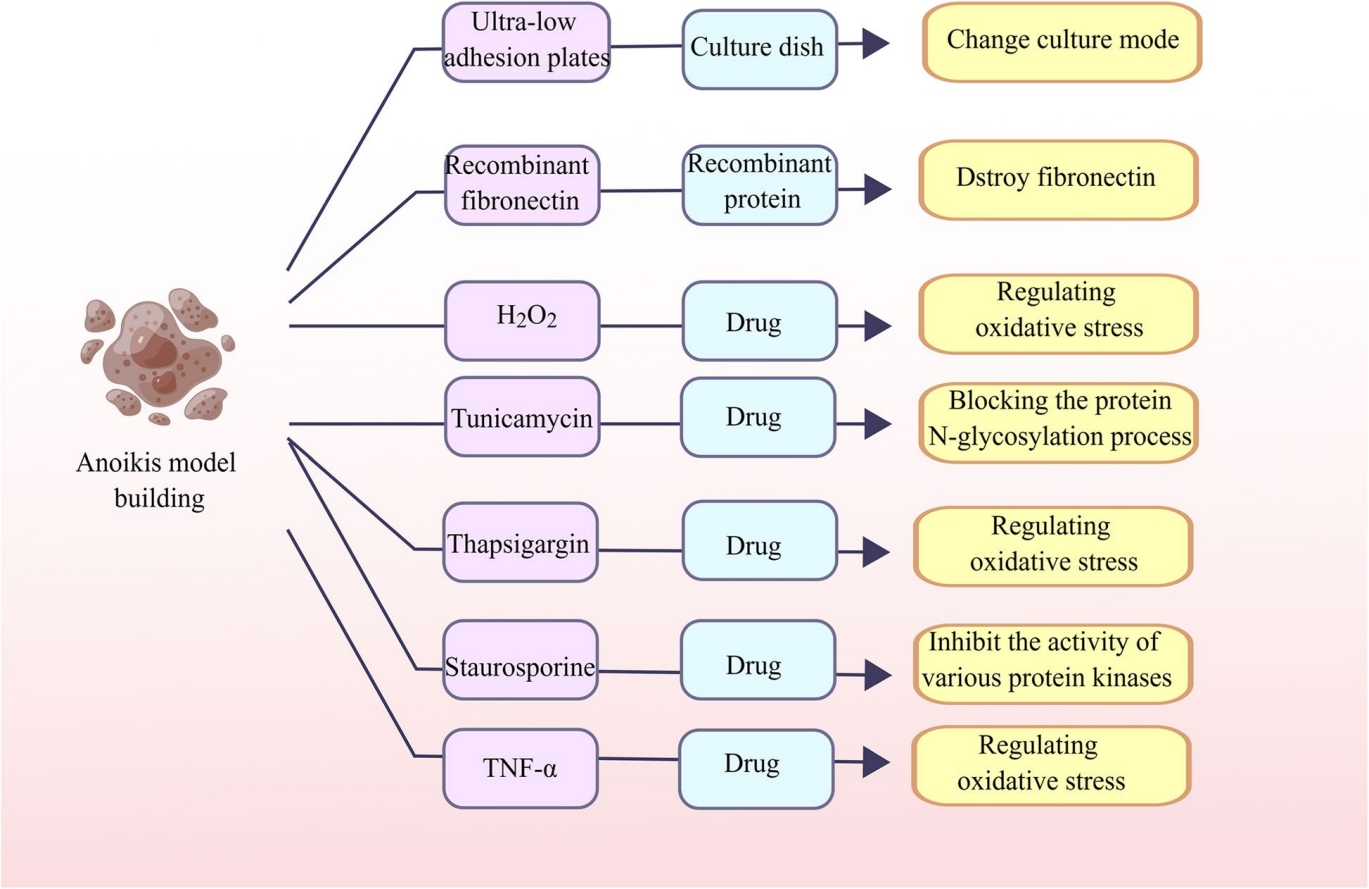
Construction of anoikis model to construct an anoikis model, different approaches can be used, including the use of specialized cell culture dishes and various drugs. Common drugs that induce apoptosis include H_2_O_2_, tunicamycin, thapsigargin, staurosporine, and TNF-α. These drugs can induce anoikis through different mechanisms, such as oxidative stress, endoplasmic reticulum stress, and protein aggregation. Therefore, the selection of these drugs should be based on specific experimental conditions and research purposes

TNF-α is an inflammatory factor that can trigger the process of cell anoikis [61] (Fig. 3).

### Death receptors mediate anoikis

#### Classification and functional mechanism of death receptors

Many studies suggest that there are multiple pathways of anoikis regulation [62], among which integrin and death receptors that mediate cell adhesion can participate in resistance to anoikis [63] Due to decreased cell adhesion, cells undergo detachment from the substrate, leading to a floating state and triggering anoikis. However, studies have also found that in the presence of TRAIL, cells lacking BAX display membrane vesiculation and detachment, indicating that TRAIL can rapidly separate cells without inducing cell death. This detachment phenomenon is similar to anoikis but not exactly anoikis, as the cells lacking Bax can reattach and survive during treatment [64]. Nevertheless, it should not be denied that the resistance to anoikis can vary among different cell types when they detach from the extracellular matrix, due to the specificity of each cell type. Tumor cells develop resistance to anoikis, which is influenced by various factors such as extracellular matrix composition, cell–cell interactions, and activation of signaling pathways. Therefore, the fate of cells towards apoptosis or survival should be discussed in a specific context. However, the mechanism of anoikis mediated by the death receptor pathway remains unknown [65]. Several studies have indicated that in colon cancer cells, the inhibition of caspase-8 activity in the extrinsic pathway leads to heightened resistance to anoikis. In contrast, the inhibition of caspase-9 does not elicit the same phenomenon, and the cells still undergo anoikis. These findings highlight the distinct roles of caspase-8 and caspase-9 in regulating anoikis in colon cancer cells [28]. Therefore, it is hypothesized that the caspase-8-mediated external pathway is more important in colon cancer cells; therefore, we speculated whether the same phenomenon is observed in other tumor cells, indicating that the death receptor is an important mechanism for anoikis. Death can be mediated by activating the death receptor pathway and caspase [66]. The activation or oligomerization of death receptors leads to the formation of a DISC and subsequent caspase activation to mediate cell death.

**Fig. 4:**
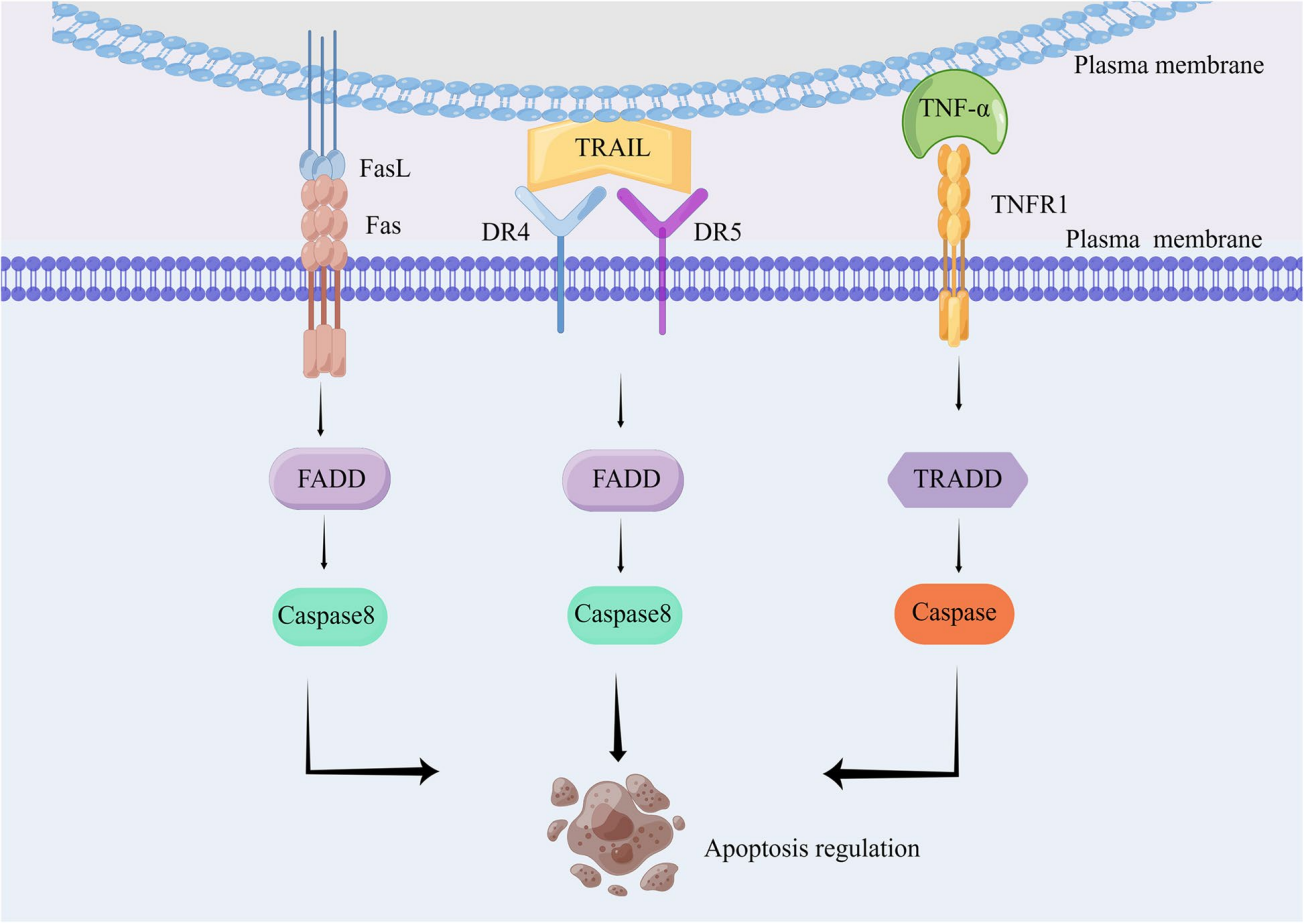
Binding of different death receptors to ligands and downstream signal transduction. Fas and FasL combine to activate downstream FADD and cause caspase activation. TNFR binds to TNFR1 to recruit TRADD, and post-translational modification of RIPK1 determines cell apoptosis. DR4 and DR5 are involved in an external pathway mediating caspase-8 activation

Death receptors are not specific organelles but a class of transmembrane proteins on cells that transmit apoptotic signals and bind to special death ligands [67]. They belong to the tumor necrosis factor receptor (TNFR) gene superfamily [68, 69], whose extracellular regions contain cysteine-rich regions, and the cytoplasmic region has a structure composed of homologous amino acid residues with proteolytic function [70, 71]. This is called the death domain (DD) [72]. The DD enables the further transmission of death signals to initiate apoptosis [73]. Caspases can also be activated by external pathways after extracellular ligands trigger cell surface death receptor activation [48]. Currently, the tumor necrosis factor (TNF) and TNFR (TNF receptor) family members are important signaling molecules involved in various biological processes in the field of life sciences. The TNFR family encompasses a group of cell surface receptors that bind to TNF family ligands. These receptors transmit signals into the cell upon ligand binding, thereby regulating diverse cellular responses. Examples of TNFR family members include Fas receptor (CD95), TNFR1/TNFR2,, DR3, and TNF-related apoptosis-inducing ligand (TRAIL) receptors (DR4 and DR5), etc. [74]. On the other hand, the TNF family consists of cytokines that play crucial roles in immune regulation, inflammation, cell survival, and cell death. Some well-known members of the TNF family include Fas ligand (FasL), TNF-α, Vascular Endothelial Growth Inhibitor (VEGI), and TRAIL [41, 75]. Fas/CD95: Fas is a cell membrane receptor protein that can bind with its ligand FasL to initiate a cell death signaling pathway called apoptosis under certain circumstances [76]. TNFR1/TNFR2 (TNF receptor 1/TNF receptor 2): TNFR1 and TNFR2 are cell membrane receptor proteins that are the main receptors for TNF-α. They can bind with TNF-α and trigger various signaling pathways [77]. DR4/DR5 (Death Receptor 4/Death Receptor 5): DR4 and DR5 are cell membrane receptor proteins that serve as receptors for TRAIL. Binding of TRAIL with DR4 or DR5 can trigger cell death signaling pathways [78]. FasL: FasL (CD95L) is a type II membrane protein and the ligand for the Fas receptor [79]. When FasL binds with Fas, it activates the Fas receptor and initiates cell death signaling pathways. TNF-α: TNF-α is a cytokine produced by immune cells. By binding with its receptors, such as TNFR1 and TNFR2, it triggers intracellular signaling pathways that regulate apoptosis and other biological processes [77]. VEGI: VEGI is an anti-angiogenic factor. TRAIL: TRAIL is a cytokine and the ligand for DR4 and DR5. Binding of TRAIL to DR4 or DR5 can initiate cell death signaling pathways [78]. Ligand binding induces receptor activation, which leads to the formation of a DISC that activates pro-caspase [80]. The complex members include junction subproteins, FADD and TNF receptor 1 associated-death domain (TRADD) are involved in the recruitment and activation of caspase-8 and the downstream executioner caspase-3. This activation cascade ultimately leads to apoptosis initiation through the formation of DISC involving death domains (DDs) [81] (Fig. 4).

#### Fas death receptors mediate anoikis

The signaling of apoptosis mediated by Fas molecules begins with the activation of death receptor molecules [82]. Fas is activated by its ligand FasL or other excitatory antibodies, resulting in the cross-linking of three Fas receptor molecules to form trimers [83]. The intracellular death function regions gather into clusters, and the corresponding death function regions of the connector protein FADD are cross-linked to the death function regions of Fas and are recruited [83]. Fas-FasL-FADD-pro-caspase-8 forms a death-inducing signal complex (DISC) by combining in series [84]. Pro-caspase-8 in DISC cleaves itself and matures into a catalytically active form, releasing its active subunits to directly activate the downstream factors caspase-3, caspase-6, and caspase-7 [85].

#### Increased transcription level of Fas

Studies have shown that increased Fas levels in anoikis subsequently induce DISC formation [86]. When Fas siRNA is used to inhibit the expression of Fas on the cytoplasmic membrane surface, the lack of Fas can influence the resistance of cells to anoikis, thus suggesting that Fas is essential for the regulation of anoikis by death receptors. These experiments proved that the activity of Fas and the formation of a death-inducing signaling complex are important for tumor metastasis [87, 88].

**Fig. 5:**
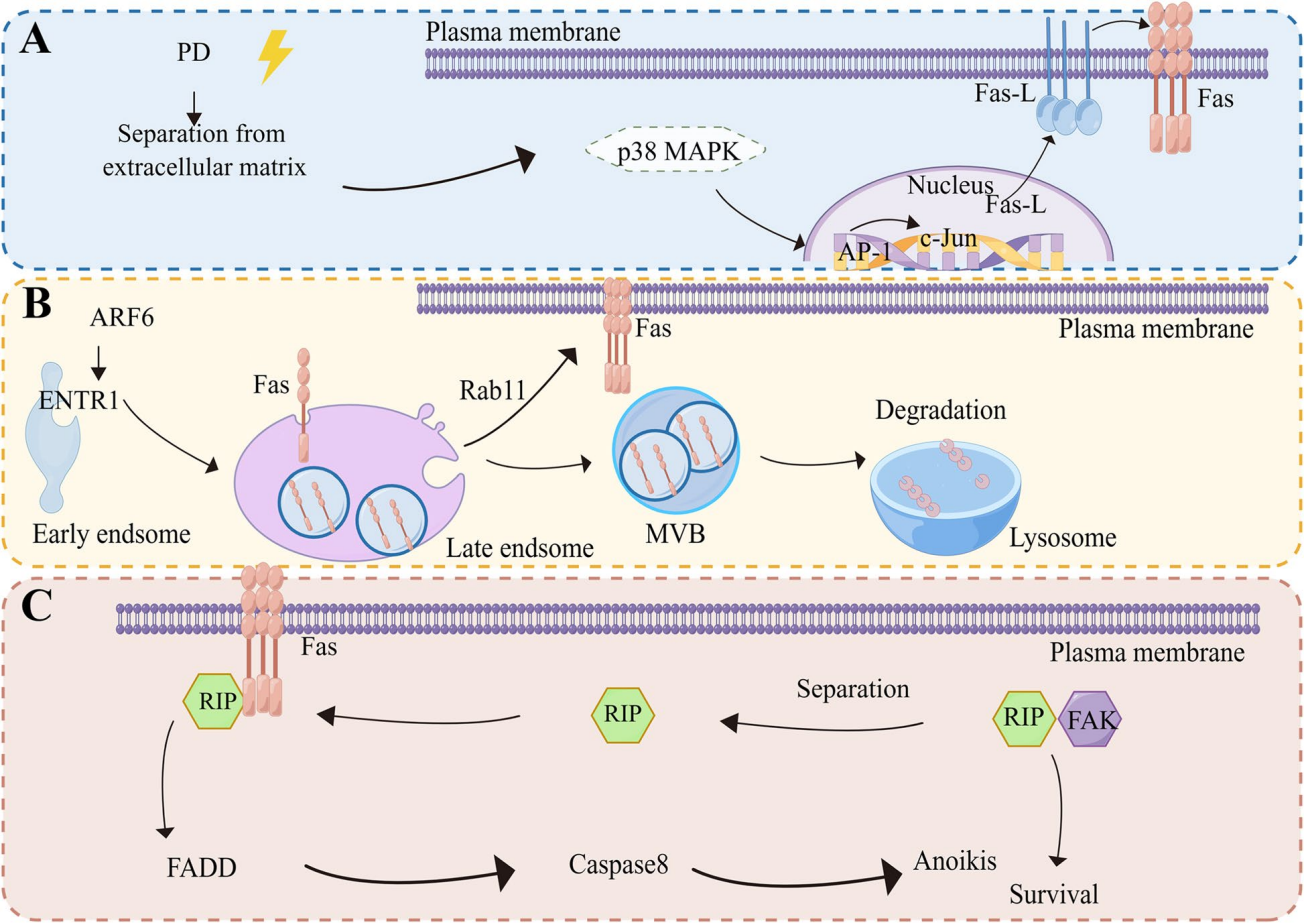
Three possible mechanisms of Fas-mediated anoikis. Elevated Fas expression levels result in the formation of downstream FADD and activation of caspase. **A** Firstly, the increase in the transcription level of Fas ligand FasL leads to the increase in the transcription level of receptor Fas and induces the formation of FADD. **B** Secondly, the increased expression level of Fas may be due to the decreased degradation of lysosomes through vesicle sorting through endocytosis, which leads to the increased accumulation of Fas in endosomes and the increased location of Fas to the plasma membrane surface dependent on Rab11-mediated recirculation and activation of downstream FADD. **C** The third is that RIP shuttles between Fas and FAK to regulate anoikis. RIP can form complexes with Fas or FAK to activate different signaling pathways under different conditions

Studies have shown that Fas-mediated death receptors cause tumor cells to undergo anoikis mainly due to two aspects: the first is the increase in Fas transcription level. Studies have shown that when platycodin is used to treat cells, the cells lose their adhesion ability, and the expression of Fas and Fas-L is increased after the loss of contact with ECM, resulting in the anoikis-like apoptotic cell death. This is due to an increase in the transcription level of the Fas-L, which subsequently causes an increase in the Fas transcription level. Activation of the formation of downstream death domain proteins and death complexes causes a series of downstream caspase shear. Experiments have shown that the increased transcription level of Fas-L induced by this drug is due to the increased entry of the AP-1 transcription factor into the nucleus, which regulates the transcription of Fas and Fas-L by promoting the activation of the P38 MAPK pathway. Death then induces the formation of a signaling complex [89] (Fig. 5A).

It has also been demonstrated in endothelial cells that dissociation-induced anoikis itself is the result of activation by the Fas pathway ligand Fas-L [88].

#### Transport of Fas in the cytoplasmic membrane

Another aspect is that a key mechanism by which cells regulate their response to specific ligands is by regulating the level of the corresponding plasma membrane receptors on the cell surface, and changes in receptor circulation may be part of the disease mechanism. Studies have shown that membrane transport of death receptors plays an important role in Fas apoptosis signaling, and effective formation of DISC occurs at the endosomal level; therefore, endocytosis of Fas is crucial. Fas exists on the plasma membrane as a pre-binding trimer, and ligands bind to Fas to induce conformational changes that allow their cytoplasmic death domains to bond with other proteins, thus forming what is known as DISC.

Sharma et al. found that the endosome associated trafficking regulator 1 (ENTR1) as a negative regulator of Fas mediated apoptosis, controls cell surface levels of Fas and Fas-mediated apoptotic signaling. Experiments have shown that ENTR1, located in early and circulating endosomes, is an important participant in Fas endocytosis by facilitating the sorting of Fas into vesicles. In the early endosome, Fas degradation towards the lysosome increased, while Fas accumulation and the Rab11-dependent cycle decreased, thus closing Fas-dependent signal transduction, regulating Fas degradation, and inhibiting apoptosis. In addition, It was also found that ENTR1 was inhibited after Fas-induced apoptosis [33].

Because some cancer cells express Fas at low surface levels, thereby escaping potential Fas-induced apoptosis, the underlying molecular mechanisms are often unclear. In this study, Fas-mediated endocytosis was suggested to induce apoptosis in downstream complex formation, and ENTR1 loss was associated with Fas-induced enhanced apoptosis sensitivity. While death receptors play a crucial role in transmitting apoptosis-inducing signals triggered by specific death ligands, we suggest that Fas endocytosis is a new mechanism by which Fas mediates anoikis. Studies have shown that ARF6, upstream of ENTR1, can positively regulate ENTR1 [90]; therefore, the possible involvement of ARF6 in regulating anoikis needs further clarification (Fig. 5B).

#### FAP-1 in regulating the membrane expression of Fas/CD95

The role of FAP-1 in regulating the membrane expression of Fas/CD95 is an important aspect to consider in the context of anoikis. FAP-1, also known as PTPN13, is a protein tyrosine phosphatase that has been shown to interact with Fas/CD95 and regulate its activity. FAP-1 can dephosphorylate Fas/CD95, leading to its internalization and degradation, thereby reducing its membrane expression. In the context of disease, the regulation of Fas/CD95 membrane expression by FAP-1 may have significant implications. Decreased expression of Fas/CD95 on the membrane can result in reduced activation of the Fas/CD95-mediated death receptor pathway, leading to decreased sensitivity to disease. On the other hand, increased expression of FAP-1 can enhance the internalization and degradation of CD95, promoting anoikis in cells that have lost contact with the extracellular matrix [91]. Although the current review does not specifically discuss the role of FAP-1 in regulating Fas/CD95 membrane expression, it is indeed an important aspect to fully understand the disease mediated by the death receptor pathway. Further research on the interaction between FAP-1 and Fas/CD95 in the context of anoikis can provide valuable insights into the mechanisms of tumor metastasis and potential therapeutic strategies.

#### Receptor-acting protein (RIP) mediates death receptor pathway based on survival signals

The loss of adhesion ability of epithelial cells from the matrix leads to decreased survival signals mediated by FAK, the largest group of integrins, leading to anoikis. Studies have shown that inhibition of FAK can promote anoikis and inhibit metastasis, whereas overexpression of FAK is associated with tumor invasiveness and metastasis. FAK transduces integrin-mediated signals that regulate cell survival and migration. Studies have shown that RIP is also involved in integrin-mediated survival signals when regulating death receptor Fas-mediated anoikis. RIP stands for receptor-interacting protein, RIP family has two members, RIP1 and RIP3. Under anoikis, RIP dissociates from FAK and forms complexes with Fas, whereas under survival conditions, RIP dissociates from Fas and forms complexes with FAK. In FAK^−/−^ cells, the reintroduction of FAK promotes survival; under anoikis conditions, these complexes decrease, and RIP-Fas complexes become more abundant [52]. It has also been reported that RIP plays a shuttling role between survival and death signaling pathways to mediate anoikis in oral cancer, revealing that SIRT3 regulates anoikis in oral cancer through a potential negative correlation with RIP. These findings suggest that RIP plays a pro-apoptotic role in anoikis by shuttling between Fas and FAK to regulate anoikis. Thus, RIP is a key signal and shuttle protein that simultaneously communicates with the integrin FAK survival signal and Fas death signal in anoikis [92]. Therefore, RIP may be an important early and upstream target for anoikis regulation (Fig. 5C).

#### TNFR1/TNFR2 death receptor mediates anoikis

Death receptors bind ligands to induce apoptosis [93]. Fas-L for Fas, TNF-α for TNFR1, and the ligands of TRAIL for DR4 and DR5 bind to each other to induce cell death via death receptors, leading to the formation of a DISC [94, 95]. This complex regulates caspase-8 activation. The binding of TNF-α to TNFR1 triggers the formation of distinct signaling complexes, leading to diverse cellular outcomes. TNF-α binds to TNFR1 and initiates the activation of the TNFR1 receptor, resulting in a series of downstream signaling events, and the death domain in its cytoplasmic tail rapidly recruits the adaptor protein TRADD [96]. TRADD, in turn, recruits TNF receptor-associated protein 2 (TRAF2), TRAF5, receptor-associated protein kinase 1 (RIPK1), linear ubiquitin chain assembly complex (LUBAC), and cellular inhibitor of apoptosis proteins cIAP1 and cIAP2 to form a signaling complex called Complex I. Complex I internalizes and transforms into the death-inducing complex, Complex II, with additional recruitment of FADD and pro-caspase8. TNFR1 activation has a number of outcomes that depend on the post-translational modification of RIPK1, a member of Complex I: polyubiquitination of RIPK1 leads to cell survival and inflammation; phosphorylation of RIPK1 prevents its interaction with FADD, leading to independent apoptosis of RIPK1. Deubiquitination of RIPK1 is beneficial for releasing RIPK1 from Complex I and binding to Complex II to induce apoptosis. Whether RIPK1 deubiquitination causes anoikis remains unclear [97].

**Fig. 6:**
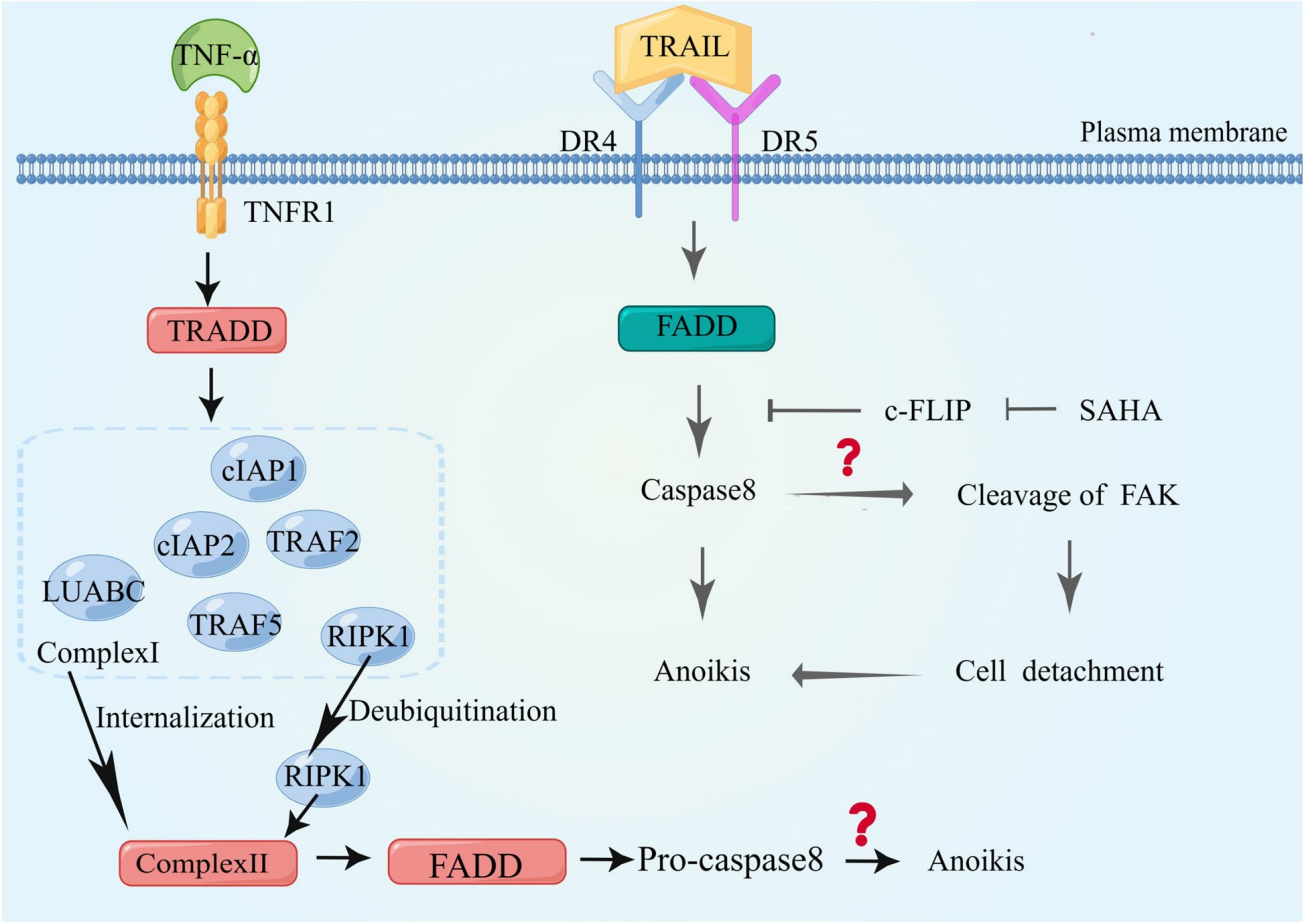
Possible mechanisms of anoikis mediated by TNFR1 and DR4/DR5. TNF-α binding to TNFR1 leads to the formation of different signaling complexes that define different cell fates. TNF-α binding to TNFR1 recruits TNF receptor-associated proteins and the death domain (TRADD). TRADD forms signal complex I with TRAF2, TRAF5, RIPK1, LUBAC, cIAP1, and cIAP2. Complex I internalizes and transforms into the death-inducing complex, Complex II, with additional recruitment of FADD and pro-caspase8. Many outcomes of TNFR1 activation depend on post-translational modification of Complex I member RIPK1; deubiquitination of RIPK1 facilitates its release from Complex I and binding to Complex II to induce apoptosis. DR5 and DR4 can participate in anoikis, but the specific mechanism remains to be clarified

It has also been shown that β-catenin induces downregulation of the apoptosis inhibitor tumor necrosis factor receptor 1 (TNFR1), and the subsequent decrease in the activity of the TNFR1 signaling mediator transcription factor NF-κB (nuclear factor-κB) triggers pro-anoikis signaling in these cells. In conclusion, the activation of TNFR1 promotes anoikis apoptosis [98] (Fig. 6).

#### DR4/DR5 death receptors mediate anoikis

SAHA, as a histone deacetylase activity inhibitor, has been shown to sensitise tumor cells to DR4/DR5 ligand TRAIL induced apoptosis [99, 100]. The study demonstrate that the combined treatment of SAHA and TRAIL induced apoptosis in mda-mb231 cells cultured on poly-HEMA, indicating a restoration of sensitivity to anoikis in these cells. This effect is associated with a significant decrease in FAK, EGFR, and phospho-ERK1/2 levels, as well as an increase in the dephosphorylated form of BimEL. Moreover, SAHA reduces the level of c-FLIP, facilitating the interaction between TRAIL and the specific death receptors DR4 and DR5, leading to subsequent activation of caspase-8. Given that the caspase-8 inhibitor z-IEDT-fmk can prevent FAK cleavage and cell detachment, it is hypothesized that caspase-8 activation may be the cause of FAK reduction and subsequent cell detachment. In summary, further experimentation is needed to validate whether combined treatment with SAHA and TRAIL induces anoikis, thereby suggesting the potential involvement of DR4/DR5 in anoikis [101] (Fig. 6).

Death receptors play a pivotal role in transmitting signals that trigger apoptosis in response to specific death ligands. These receptors are instrumental in initiating the process of apoptosis by facilitating the transmission of signals that induce cell death in response to specific ligands [102], and Rytomaa et al. [103] showed that external ligand activation of death receptors is not required for caspase activation or apoptosis during disease development. Knockdown of DR5 or TRAIL inhibits anoikis, whereas exogenous TRAIL or Fasl do not increase anoikis. Therefore, it is suggested that the DR5 receptor mediates the apoptotic pathway through the exogenous death receptor pathway and is required for anoikis induction, as antibodies against soluble TRAIL do not alter anoikis induction [28].

Some studies have suggested that DR5 receptor-mediated apoptosis through the exogenous death receptor pathway is the core mechanism leading to anoikis. DR5 is cross-activated by soluble and membrane-bound TRAIL ligands but not by soluble non-crosslinked ligands, whereas DR4 is activated by soluble and membrane-bound ligands [104, 105]. It can be seen that membrane-bound ligands promote death and participate in the formation of downstream death complexes during suspension culture [106]. However, the contribution of membrane-bound ligands and receptor oligomers to initiating the death response remains to be elucidated.

## Conclusion

Anoikis is a vital process that plays a crucial role in maintaining tissue stability, regulating cell growth, preventing abnormal cell adhesion to the ECM, and influencing various aspects of body development, tissue homeostasis, and disease progression. This includes its significant involvement in tumor metastasis. This review summarizes the possible mechanisms of anoikis mediated by the death receptor pathway and introduces three examples of the FAS-dependent death receptor pathway. TNFR1 recruits the TRADD-mediated death receptor pathway to cause anoikis, and DR4 and DR5 cause anoikis to some extent; however, the exact mechanism needs further elucidation. Further study on the phenomenon of anoikis or anti- anoikis is helpful to further reveal the specific mechanism of tumor metastasis. This review provides new ideas and discussions for studying anoikis through the death receptor pathway, it also provides a new direction for the clinical treatment of metastatic tumors. There is substantial literature indicating that the main pathways involved in anoikis are associated with BCL2 members and the intrinsic pathway. However, evidence also suggests that death receptors, such as Fas, TNFR1, DR4, and DR5, can mediate anoikis. We have reviewed the potential mechanisms of anoikis mediated by the death receptor pathway, and understanding the role of death receptors in anoikis can provide valuable insights into tumor metastasis mechanisms and potentially lead to the development of new therapeutic strategies. It is undeniable that there is limited literature regarding the connection between death receptors and anoikis, which suggests the potential for further research in this area. Therefore, further research is needed in the future to fully understand the contribution of death receptors to disease.

## Data Availability

Not applicable.
